# Corrosion Behavior of MgTiZn and Mg_4_TiZn Alloys After Ball Milling and Subsequent Spark Plasma Sintering

**DOI:** 10.3390/ma18143279

**Published:** 2025-07-11

**Authors:** Alexander Helmer, Rahul Agrawal, Manoj Mugale, Tushar Borkar, Rajeev Gupta

**Affiliations:** 1 Department of Materials Science and Engineering, North Carolina State University, Raleigh, NC 27606, USA; rkagrawa@ncsu.edu; 2 Mechanical Engineering Department, Cleveland State University, Cleveland, OH 44115, USA; m.mugale@vikes.csuohio.edu (M.M.); t.borkar@csuohio.edu (T.B.)

**Keywords:** magnesium alloys, multi-principal element alloys, corrosion, microstructure, hardness, stiffness

## Abstract

Magnesium-containing multi-principal element alloys (MPEAs) are promising for lightweight applications due to their low density, high specific strength, and biocompatibility. This study examines two Mg-Ti-Zn alloy compositions, equal molar MgTiZn (TZ) and Mg_4_TiZn (4TZ), synthesized via ball milling followed by spark plasma sintering, focusing on their microstructures and corrosion behaviors. X-ray diffraction and transmission electron microscopy revealed the formation of intermetallic phases, including Ti_2_Zn and Mg_21_Zn_25_ in TZ, while 4TZ exhibited a predominantly Mg-rich phase. Potentiodynamic polarization and immersion tests in 0.1 M NaCl solution showed that both alloys had good corrosion resistance, with values of 3.65 ± 0.65 µA/cm^2^ for TZ and 4.58 ± 1.64 µA/cm^2^ for 4TZ. This was attributed to the formation of a TiO_2_-rich surface film in the TZ, as confirmed by X-ray photoelectron spectroscopy (XPS), which contributed to enhanced passivation and lower corrosion current density. Both alloys displayed high hardness, 5.5 ± 1.0 GPa for TZ and 5.1 ± 0.9 GPa for 4TZ, and high stiffness, with Young’s modulus values of 98.2 ± 11.2 GPa for TZ and 100.8 ± 9.6 GPa for 4TZ. These findings highlight the potential of incorporating Ti and Zn via mechanical alloying to improve the corrosion resistance of Mg-containing MPEAs and Mg-based alloys.

## 1. Introduction

Magnesium (Mg) alloys have emerged as strong candidates for applications in automotive, aerospace, and medical devices because they offer the lowest density among structural metals, high specific strength, and biocompatibility [1,2,3]. The high strength-to-weight ratio of Mg alloys provides economic and environmental benefits, as weight reductions in vehicles can significantly lower fuel consumption and emissions, aligning with recent global sustainability regulations and initiatives [1,4].

Despite these advantages, Mg alloys exhibit poor corrosion resistance, particularly in aggressive chloride environments [5,6,7]. This rapid corrosion is often accelerated by microstructural features, such as secondary phases and impurities [8]. Thus, the development of Mg-based alloys with improved corrosion resistance remains a critical area of research. To address this issue, alloying strategies that balance corrosion resistance and mechanical performance are essential.

One promising design strategy involves the development of multi-principal element alloys (MPEAs), which incorporate multiple elements in equiatomic or near-equiatomic ratios to achieve property combinations beyond those achievable in conventional binary or ternary systems. While this concept has been widely explored in high-entropy alloys, its application to Mg-rich systems remains limited and may offer new pathways for improving corrosion resistance, mechanical performance, and maintaining a low density. While prior studies have investigated Mg-Ti, Mg-Zn, and Zn-Ti binary alloys individually, the combined effects of Ti and Zn in bulk Mg-rich multi-element systems remain poorly understood, particularly with respect to phase stability, passivation behavior, and mechanical properties.

Among these systems, titanium (Ti) and zinc (Zn) have been reported to enhance the corrosion resistance of Mg alloys [9,10] by promoting the formation of intermetallic phases that act as corrosion barriers [11,12]. In combination with Zn, Ti can form stable intermetallic compounds, such as Ti_2_Zn and TiZn_16_, which provide a shielding effect and limit the progression of corrosion fronts [11,13]. In contrast, micro-galvanic effects between intermetallics and the Mg matrix, arising from electrochemical potential differences, can accelerate localized corrosion [14,15]. For example, Ti rich intermetallics may act as cathodic sites, enhancing the localized anodic dissolution of Mg [2,10]. This makes the spatial distribution, morphology, and size of these phases critical for determining corrosion performance [15]. Consequently, Mg-Ti-Zn alloys are promising for applications requiring both biocompatibility and corrosion resistance, especially in medical devices, where controlled degradation minimizes toxic byproduct formation [11,13]. In addition to corrosion resistance, these alloys must also maintain sufficient hardness and strength for load-bearing applications, motivating strategies that can simultaneously improve both properties.

Several studies have explored Mg-Zn, Mg-Ti, and Zn-Ti alloys for their potential to improve corrosion resistance and mechanical performance [9,11,13,14,16]. Mg-Zn alloys have been shown to promote grain refinement and ZnO-rich surface films, with corrosion current densities (i_corr_) typically ranging from 10 to 100 µA/cm^2^ in NaCl solutions, although their poor hardness (less than ~1 GPa) limits their broader implementation [16]. Ti additions to Mg offer excellent corrosion resistance, often reducing i_corr_ to the range of 1–10 µA/cm^2^, but are hindered by poor solubility in Mg and higher cost [9,10]. However, sputtered Mg-Ti films have demonstrated passivation via TiO_2_-rich layers and reduced current densities, although this line of study lacks bulk validation [17]. Binary Zn-Ti alloys have shown promising corrosion resistance (e.g., ~2 µA/cm^2^) and improved surface passivation due to Ti rich phases, but still face challenges in balancing degradation rate with mechanical robustness [13]. Recent work by Mukhtar et al. demonstrated that mechanical alloying followed by compaction and sintering can successfully produce bulk Mg-Ti-Zn alloys with refined nanostructures and enhanced mechanical properties [18]. However, the corrosion behavior was not evaluated, leaving open the question of how such processing routes affect the electrochemical performance in these systems.

In contrast, the present work uses a multi-principal element alloying strategy combined with ball milling and spark plasma sintering to produce bulk Mg-Ti-Zn alloys with engineered microstructures, enabling new insights into corrosion behavior, intermetallic phase stability, and surface film formation. Most previous studies used casting or sputtering, which limits compositional control and microstructural refinement. The current study overcomes these constraints by employing a powder metallurgy approach to produce bulk alloys with engineered microstructures.

Recent alloy development strategies have focused on MPEAs to exploit the synergistic effects of numerous elements [19,20]. In this study, Mg-containing MPEAs were investigated, with the Mg content systematically varied while Ti and Zn were held constant in a molar ratio. The two compositions, MgTiZn (equimolar, TZ) and Mg_4_TiZn (4TZ), enable the exploration of how increased Mg content affects the microstructure and corrosion, while highlighting the role of Ti and Zn in passivation and strengthening.

The addition of Ti and Zn to Mg is challenging due to their limited solid solubility in Mg [21]. High-energy ball milling (HEBM) and spark plasma sintering (SPS) have been reported to enhance the properties of magnesium-based alloys, particularly those containing alloying elements such as Ti and Zn [9,15,22,23]. HEBM, a form of mechanical alloying, enables microstructural homogenization, reduces grain size, and promotes the formation of new phases, including those far from equilibrium [24]. This refinement process aids in extending the solid solubility of alloying elements within the magnesium matrix, resulting in enhanced mechanical properties and corrosion resistance [25,26,27,28].

Spark plasma sintering complements HEBM by consolidating the milled powder under controlled conditions, thus maintaining the refined microstructure achieved during milling [23]. SPS uses rapid and localized heating, which reduces the risk of grain growth that typically occurs in conventional sintering [29]. SPS preserves the nanocrystalline and non-equilibrium phases formed during ball milling [22,23,27]. Studies have shown that alloys processed using a combination of ball milling and SPS exhibit improved densification, finer grain size, and reduced porosity, which contribute to their structural integrity in harsh environments [23,29,30,31,32].

The aim of the present work is to investigate the influence of systematic variations in Mg content on the microstructure, mechanical properties, and corrosion behavior of these alloys. By characterizing the resulting intermetallic phases, grain refinement, and passive film formation, this study identified how processing and composition synergistically affect the corrosion resistance and mechanical performance. This work offers new insights into the design of corrosion-resistant, high-strength materials and represents a novel extension of compositionally complex alloy design to Mg-rich systems.

## 2. Materials and Methods

### 2.1. Materials

Individual metallic powders of Mg (99.8%), Ti (99.99%), and Zn (99.9%) were obtained from Alfa Aesar (Haverhill, MA, USA), Sigma-Aldrich (St. Louis, MO, USA), and Goodfellow (San Angelo, TX, USA) with particle sizes ranging from 44 to 150 µm. For mechanical alloying, the powders were prepared in two compositions: (1) an equal molar alloy of Mg, Ti, and Zn, referred to hereafter as TZ, and (2) Mg_4_TiZn, referred to hereafter as 4TZ; the exact compositions are shown in Table 1.

The powders were placed in separate stainless steel bowls along with stainless steel balls (10 mm diameter) and 1.5 wt.% stearic acid to prevent sticking. Stearic acid was added as a process control agent to reduce cold welding, prevent excessive agglomeration, and promote uniform particle dispersion during high-energy milling [24]. A charge of 20:1 was used, with 10 g of powder placed in each bowl. The bowls were loaded and unloaded in an inert argon atmosphere to avoid atmospheric contamination. Each bowl was placed on a Fritsch Pulverisette 4/5 and milled at 280 RPM for 100 h, with a 30 min rest after each hour of milling to avoid overheating the alloys. Both bowls were opened in a glovebox, and the agglomerates were broken after 2, 4, 8, 16, 32, 64, and 100 h. Final milling was performed at 280 RPM for one minute to obtain a fine powder of each alloy composition.

Spark plasma sintering (SPS) (SPS 10-3, Thermal Technologies, Blythewood, SC, USA) was used to consolidate the mechanically alloyed powders under a uniaxial pressure of 60 MPa at a temperature of 450 °C for 5 min with a heating rate of 100 °C/min. The mechanically alloyed powder was placed inside a 10 mm diameter graphite die, and graphite foil was placed between the powder and the die and punches to avoid any reaction between the powder and the die and prevent sticking. The resulting SPS specimens had a diameter of approximately 10 mm and a thickness of 7 to 8 mm.

### 2.2. Characterization

#### 2.2.1. X-Ray Diffraction

The mechanically alloyed powders, along with the SPS samples, were analyzed using a Rigaku SmartLab X-ray Diffractometer with a Cu-K_*α*_ radiation source (λ = 1.541862 Å), operating at 40 kV and 44 mA. Data were collected by scanning each alloy over a 2θ range of 20° to 80°, with a scan speed of 2.0 degrees per minute and a step size of 0.02 degrees. The samples were ground flat using 1200-grit SiC paper and polished to 0.05 µm, as detailed in the microscopy section. X’Pert HighScore Plus version 5.2 was used for background subtraction, Cu-K_α2_ removal, peak fitting, and cross-referencing with known diffraction spectra.

#### 2.2.2. Scanning Electron Microscopy

For scanning electron microscopy (SEM) and energy dispersive X-ray spectroscopy (EDXS), the samples were polished to a 0.05 µm finish.

SEM imaging was performed using a Hitachi UHR SU8700 (Hitachi, Tokyo, Japan), with beam voltages adjusted between 2 and 20 kV. EDXS analysis was performed using an Oxford spectrometer and an insertable backscattered electron detector operating at 20 kV with high current. The acquired data, including EDXS maps, area scans, point analyses, and line spectra, were processed and analyzed using the Oxford AZtec software version 6.2.

#### 2.2.3. Scanning Transmission Electron Microscopy (STEM)

For focused ion beam (FIB) milling, a thin layer (~0.3 µm) of Pt was first deposited over a 10 µm × 3 µm area using a 2 kV electron beam to prevent surface damage. Subsequently, a 2 µm-thick Pt layer was deposited on top using a 30 kV ion beam. The area surrounding the Pt-deposited region was milled using a 30 kV ion beam. The two side surfaces of the lamella were cleaned, and an inverted J-cut was made to prepare it for transfer onto the sample grid. At this stage, the lamella thickness was approximately 2 µm. Once on the grid, the lamella was further thinned and polished, with the ion beam voltage progressively reduced from 15 to 5 kV for both the front and back surfaces. The final TEM sample was a 5 µm × 5 µm lamella with a thickness of approximately 80 nm. Selected-area electron diffraction (SAED) patterns and bright-field imaging were obtained using an FEI Talos (Thermo Fisher Scientific, Waltham, MA, USA) microscope operated at 200 kV in the micro-diffraction mode. Elemental distribution was obtained in scanning transmission electron microscopy (STEM) mode using a Super-X EDS detector (Thermo Fisher Scientific, Waltham, MA, USA) while simultaneously capturing high-angle annular dark field (HAADF) images.

#### 2.2.4. Nanoindentation

Nanoindentation was conducted using a Bruker Hysitron TI980 Triboindenter (Bruker, Billerica, MA, USA) with a 1000 µN load, 10-s hold, and 20-s ramp times. A minimum of 10 measurements were obtained from different regions of each sample. Prior to nanoindentation, the samples were ground to 1200 grit with SiC grinding paper using ethanol as a lubricant and polished to 0.05 µm.

#### 2.2.5. X-Ray Photoelectron Spectroscopy

To analyze the surface of both alloys after a 5 min immersion in 0.1 M NaCl solution, X-ray photoelectron spectroscopy (XPS) was performed. The specimens were polished to a 0.05 µm finish before immersion. XPS data were acquired using a SPECS system with a PHOIBOS 150 spectrometer (SPECS GmbH, Berlin, Germany). Spectra were collected from a ~78 mm^2^ area, with a 24 eV bandpass energy for the survey and 20 eV for the high-resolution spectra. The spectra were processed using the CASA XPS software version 2.3.26PR1.0 for atomic concentration quantification. The vacuum chamber pressure was maintained at 1.33 × 10^−6^ Pa, and the binding energies were corrected to the C 1s peak at 285.0 eV.

### 2.3. Corrosion

The corrosion behavior was evaluated using potentiodynamic polarization (PDP) tests with a Biologic VMP-300 potentiostat (BioLogic, Seyssinet-Pariset, France). The sample surfaces were prepared by grinding with 1200-grit SiC sandpaper, using ethanol as a lubricant. A conventional three-electrode setup was employed in a flat cell from Princeton Applied Research, with a platinum mesh as the counter electrode and a saturated calomel electrode (SCE) as the reference. All PDP tests were conducted in a 0.1 M NaCl solution at room temperature under ambient air. The open-circuit potential (OCP) was monitored for 20 min before the polarization. Potential scans were started 200 mV below the OCP, increasing at a rate of 1 mV/s until an anodic current density of 1 mA/cm^2^ was reached. Each corrosion test was performed in triplicate. A 0.1 M NaCl solution was selected based on its common usage in corrosion studies of magnesium alloys, enabling direct comparison with existing literature while also allowing a clearer resolution of corrosion mechanisms at lower ionic strengths [28,33].

Immersion tests were performed in a 0.1 M NaCl solution at room temperature using a 500 mL beaker open to ambient air for 24 h. The OCP was tracked using a potentiostat against an SCE as the reference. A VM-100 Digital Stereo Microscope was used for optical microscopy, and in situ immersion imaging was automated using Python 3.12, capturing bursts of three images at regular intervals. The samples were mounted in epoxy, encasing the contact point with a copper wire affixed to the back, ensuring that only the prepared surface was exposed to the solution. The surface of each sample was polished to 0.05 µm using the same method described in the microscopy section.

## 3. Results and Discussion

### 3.1. Microstructural Characterization Using SEM, XRD, and S/TEM

**Figure 1:**
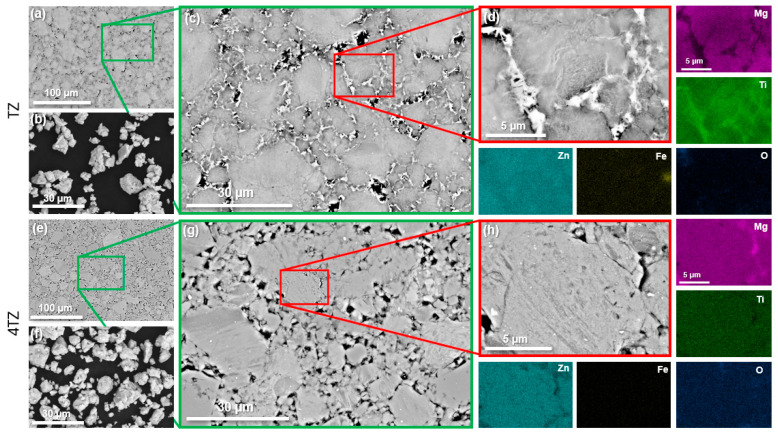
SEM micrographs of TZ (top row) and 4TZ (bottom row); (**a**) is a BSE image of the microstructure of TZ after spark plasma sintering, (**b**) is a BSE of the powder produced from milling, (**c**) shows a higher magnification BSE image of (**a**), and (**d**) is a magnified region of (**c**) with EDXS area maps, (**e**) is a BSE image of 4TZ after spark plasma sintering, (**f**) is a BSE of the powder after milling, (**g**) is an increased magnification BSE of (**e**), and (**h**) is a higher magnification BSE image of a region of (**g**) with EDXS area maps.

The microstructures of both alloys produced by SPS, along with the ball-milled powders, are shown in Figure 1. Low-magnification SEM images (Figure 1a,e) revealed numerous micropores in both alloys. High-magnification images (Figure 1c,g) show that the regions near the micropores were different in the two alloys. EDXS analysis revealed Mg-lean and Ti-enriched regions near micropores in the TZ alloy (Figure 1d). Trace amounts of Fe were also detected in the TZ alloy but not in the 4TZ alloy. This contamination likely originated from the stainless-steel balls and milling jar. Such Fe contamination is commonly reported in long-duration milling and may contribute to localized corrosion in Mg-based systems due to the cathodic nature of Fe relative to Mg [33,34]. However, the Fe concentration observed in the TEM was below 3 at.% and limited to isolated regions. Ti rich phases were absent in the 4TZ alloy; however, regions with higher Mg content and lower Zn content were observed. The observed microstructural differences between the alloys highlight the combined influence of HEBM, SPS, and composition. Localized Joule heating at the particle boundaries during SPS likely yields elevated temperatures in these areas, accelerating the formation of equilibrium phases [35] such as intermetallics [29,30]. Moreover, the limited mutual solubility between Mg and Ti, along with the lack of intermetallic compound formation between them [36], further explains why Mg segregated from the region, leading to the formation of a Ti and Zn rich phase.

**Figure 2:**
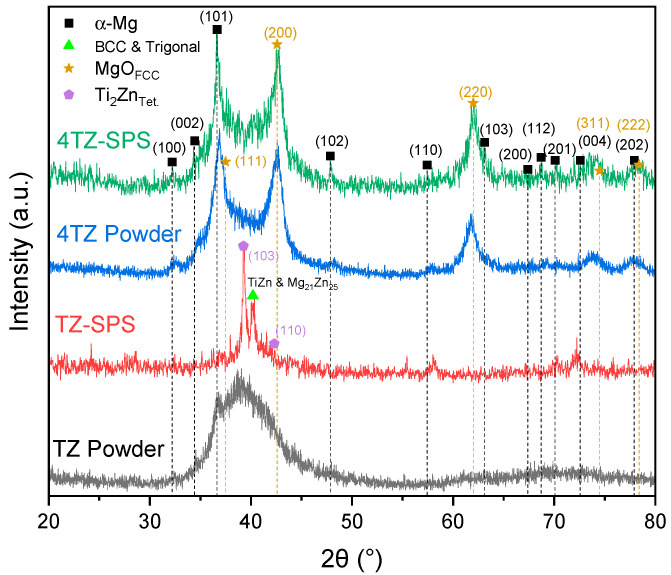
XRD of TZ powder after milling (black), followed by TZ-SPS (red), third from the bottom (blue) milled 4TZ powder, and the topmost (green) 4TZ-SPS. The background and satellite peaks were subtracted for analysis but were included for presentation. The peaks for α-Mg (PDF# 00-004-0770) are shown as black squares, the green triangle indicates a peak that is the primary peak of both BCC TiZn (PDF# 04-007-4111) and trigonal Mg_21_Zn_25_ (PDF# 04-011-0752), peaks of FCC MgO (PDF# 04-016-2776) are shown as stars, and peaks of tetragonal Ti_2_Zn (PDF# 04-004-3986) are shown as hexagons.

The X-ray diffraction (XRD) patterns (Figure 2) revealed complex phase compositions for both TZ and 4TZ alloys, with notable differences between the as-milled powders and their corresponding SPS samples. All patterns showed low intensity and significant peak broadening, which are typical features of HEBM materials. These characteristics result from grain refinement, lattice strain, and partial amorphization, which are introduced during the milling process [24]. Although background subtraction was attempted, it did not significantly improve the signal-to-noise ratio and was therefore omitted to avoid overprocessing and loss of minor features. The TZ powder exhibited only the main peak corresponding to the Mg HCP (101) plane, along with a broad peak in the region where the primary peaks of Zn, Ti, Ti_2_Zn, and Mg_21_Zn_25_ were expected. This broad peak in the TZ powder may be attributed to peak overlap, amorphization, or lattice strain. After SPS, the peak broadening disappeared, and distinct peaks corresponding to Ti_2_Zn and Mg_21_Zn_25_ became evident.

The 4TZ powder showed α-Mg peaks along with peaks associated with the FCC phase, matching with magnesium oxide (Figure 2). The high MgO content observed in the 4TZ samples (both powder and SPS) was in contrast to that in the TZ samples, where MgO was not apparent. The significant presence of MgO in the milled 4TZ powder suggests oxygen contamination during the milling process, which is consistent with the observations of Grosjean et al. [28]. The high reactivity of magnesium promotes rapid oxidation under high-energy milling conditions, which repeatedly fractures and exposes fresh Mg surfaces. As reported in similar studies, oxygen incorporation likely occurred early in the milling process, potentially from residual air or surface oxides on the milling media, resulting in MgO levels that were stabilized over time [28,37]. The presence of other elements in the Mg and MgO phases cannot be ruled out; Ti and Zn may have dissolved within the MgO and Mg matrices, potentially forming mixed oxide structures or complex oxide phases, as observed in [38]. This hypothesis could explain the absence of distinct Zn or Ti peaks, as their presence may have been masked by a broad MgO-related peak.

**Figure 3:**
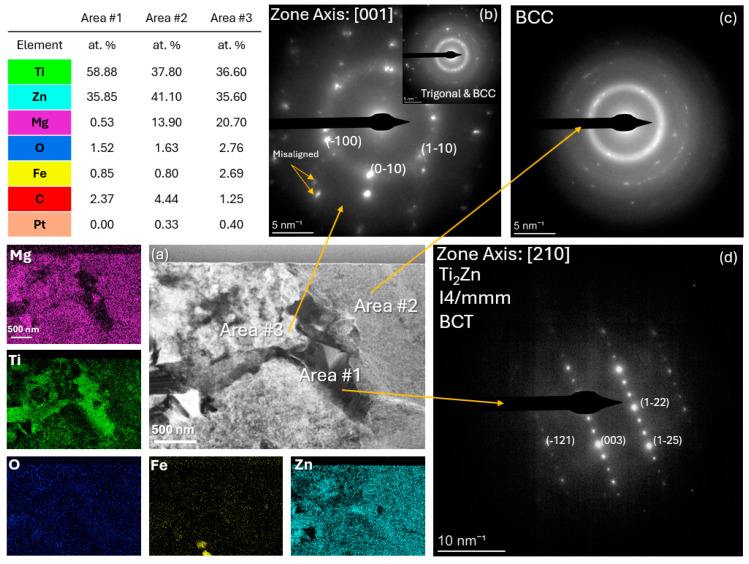
STEM of TZ (**a**) shows a HAADF with EDXS, (**b**) is a selected area diffraction pattern and ring pattern of Area #3, (**c**) is a ring pattern from Area #2, and (**d**) is a selected area diffraction pattern from Area #1.

**Figure 4:**
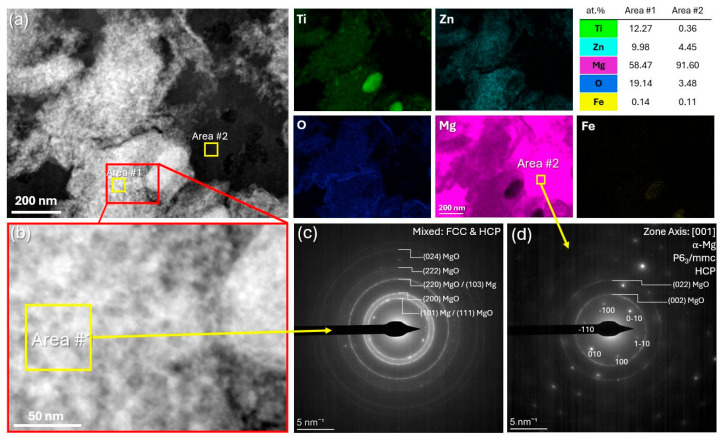
STEM of 4TZ (**a**) HAADF image of an area of the cross-section along with EDXS on the right, (**b**) enlarged HAADF image of 4TZ, (**c**) indexed SAED pattern of Area #1 showing rings for MgO (FCC) and some overlap of Mg (HCP), and (**d**) indexed SAED pattern from Area #2 showing the [001] zone axis of α-Mg.

Figure 3 and Figure 4 show the high-angle annular dark-field (HAADF) images along with the elemental distribution for the TZ and 4TZ alloys produced by SPS. Three areas with different compositions and crystal structures were observed for the TZ sample (Figure 3). A Ti rich, Mg-free area #1 was indexed to a body-centered tetragonal (BCT) Ti_2_Zn phase. Area #2 exhibited a more equal distribution of Ti and Zn, and the corresponding diffused ring pattern suggested a BCC crystal with very fine grains. The spots in the SAED pattern from Area #3 match the [001] zone axis of trigonal Mg_21_Zn_25_, while the ring pattern matches the BCC phase. Compositionally, area #3 contained a significantly higher concentration of Zn and a moderate amount of Mg. The phase composition of TZ appeared to be a mixture of Mg, tetragonal, and trigonal phases. EDXS analysis suggests that these phases are doped with other alloying elements.

Figure 4a shows a HAADF image of 4TZ with two distinct areas marked: Area #1 and Area #2. The EDXS analysis indicated the presence of Ti and Zn, with Mg as the dominant element in the bright phase (Area #1). Oxygen was also detected, suggesting the formation of oxides or a high amount of O at the interstitial sites. Figure 4b shows an enlarged HAADF image of Area #1, showing finer structural details. Figure 4c presents an indexed SAED pattern of Area #1, revealing ring patterns attributed to MgO (FCC) and some overlap with α-Mg, indicating that a mixture of oxide and metallic phases was present in this region. In Figure 4d, the SAED pattern from Area #2 is shown, indexed to the [001] zone axis of α-Mg, highlighting the HCP crystal structure of magnesium.

A combination of XRD, SEM, and TEM revealed the presence of multiple phases in both alloys. Throughout this work, the term intermetallic phase refers to chemically ordered compounds, such as Ti_2_Zn and Mg_21_Zn_25_, confirmed by SAED, while solid solution describes elemental distributions within Mg-rich regions detected via STEM-EDXS. TZ appeared to be composed of Mg, along with tetragonal and trigonal phases. 4TZ contained Ti and Zn containing MgO phases and Mg with a dispersion of fine Ti and Zn rich phases, which were beyond the resolution of XRD.

### 3.2. Hardness

**Figure 5:**
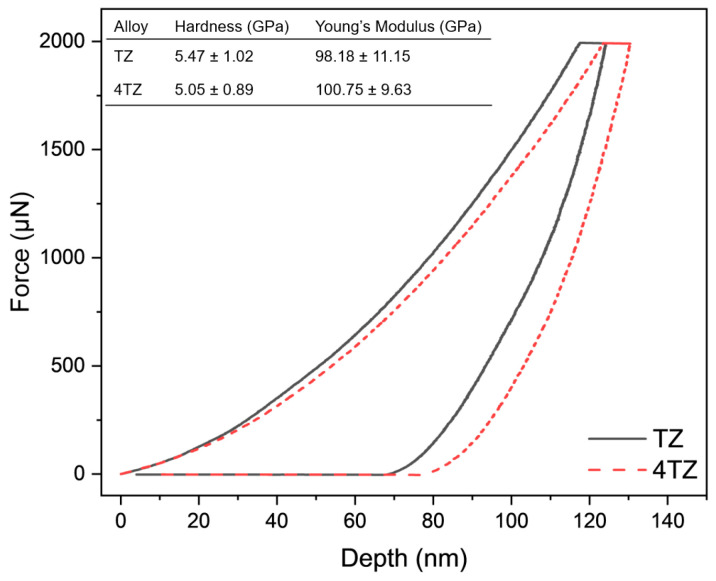
The force vs. displacement curves from nanoindentation, along with the hardness and Young’s modulus calculated from nanoindentation.

The force displacement curves for TZ and 4TZ are shown in Figure 5, along with the extracted hardness and Young’s modulus values. Both alloys exhibited high hardness, 5.5 ± 1.0 GPa for TZ and 5.1 ± 0.9 GPa for 4TZ, despite the significantly higher Mg content in 4TZ than in TZ. This can be attributed to the severe lattice distortion and high defect density introduced by ball milling, which offsets the softening effect of the Mg. As shown in the loading curves, TZ penetrated less deeply under the same applied force, reflecting its higher hardness and resistance to plastic deformation. During unloading, the slightly steeper slope of the 4TZ curve reflects its marginally higher average Young’s modulus (100.8 ± 9.6 GPa) compared to that of TZ (98.2 ± 11.2 GPa), although the values overlapped within error.

Notably, these curves underscore the enhanced mechanical performance of both alloys relative to conventional Mg systems, with hardness values approximately four times higher than those of commercial Mg alloys [39] and elastic moduli approaching those of commercial Ti alloys [28,37]. The high modulus of the Mg-rich 4TZ alloy suggests its potential for structural applications where the stiffness-to-weight ratio is critical. For more context, Zn-Ti alloys typically exhibit hardness values in the range of 0.3–3.8 GPa and Young’s moduli of 4.3–26.8 GPa [11,12,13], while Mg alloys such as AZ31 and WE43C show hardness values of 0.6–0.93 GPa and moduli around ~45 GPa [40,41,42,43]. The significantly higher hardness and modulus observed in TZ and 4TZ reflect the microstructural refinement and solid-solution strengthening induced by ball milling and SPS processing. A broader comparison of the mechanical properties of conventional Zn, Ti, and Mg-based alloys is presented in Table 2.

### 3.3. Corrosion Behavior

**Figure 6:**
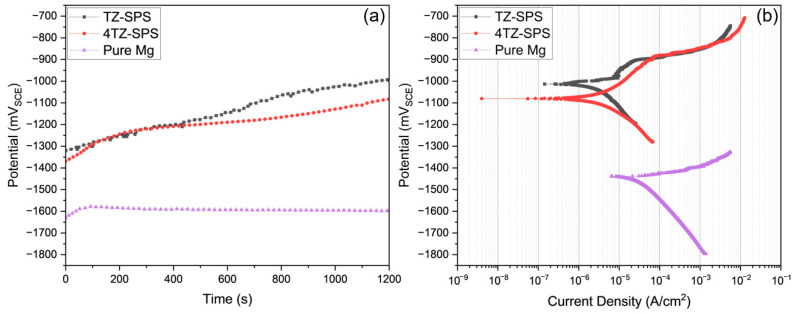
(**a**) Potential vs. time plots and (**b**) potentiodynamic polarization curves of TZ-SPS (black square), 4TZ-SPS (red circle), and cast Pure Mg (purple triangle) in 0.1 M NaCl.

The representative open-circuit potential (OCP) versus time and potentiodynamic polarization (PDP) curves for TZ (black) and 4TZ (red) are shown in Figure 6a and Figure 6b, respectively. The OCPs of the TZ and 4TZ alloys were similar but much higher than those of the Mg alloys. The PDP curves show that both the TZ and 4TZ alloys exhibit passivity, with slight differences in their anodic branches. TZ alloys showed a rapid increase in anodic current density, which stabilized at ~30 mV above the corrosion potential (E_corr_). The anodic current density for the 4TZ alloy was slightly higher than that for the TZ alloy, which could be attributed to the higher Mg content. The average corrosion potential for TZ was −972.38 ± 78.08 mV_SCE_, while 4TZ had an average potential of −1078.39 ± 2.31 mV_SCE_. The corrosion current densities, calculated from the Tafel slopes, were 3.65 ± 0.65 µA/cm^2^ for TZ and 4.58 ± 1.64 µA/cm^2^ for 4TZ.

Table 3 presents a comparison of the corrosion potentials and corrosion current densities of the TZ and 4TZ alloys with values reported in the literature for various Zn, Ti, and Mg-based alloys. Although binary Zn-Ti alloys often exhibit higher corrosion potentials, their current densities are typically an order of magnitude greater than those of TZ and 4TZ. In contrast, common Mg alloys such as ZK60 and AZ31 exhibited significantly more negative E_corr_ values and substantially higher i_corr_ values. The performance of TZ, in particular, is favorable, combining a low corrosion current density with a more noble potential. This highlights the potential advantages of the MPEA design strategy in achieving improved corrosion resistance while maintaining lightweight compositions.

**Figure 7:**
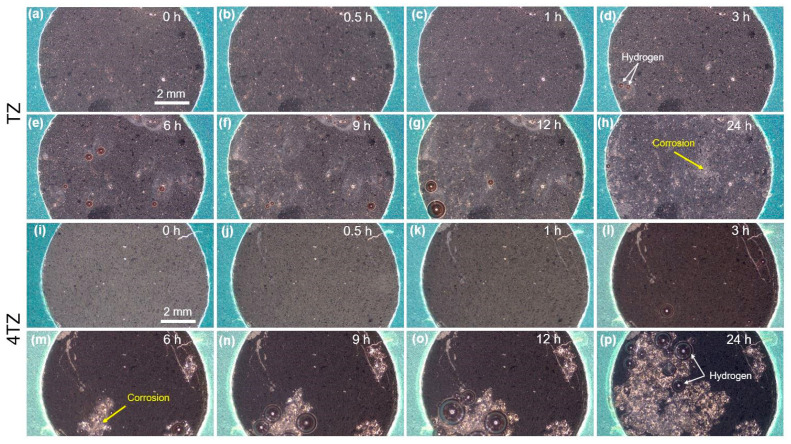
Optical microscopy images of TZ in the top two rows (**a**–**h**) at 0, 0.5, 1, 3, 6, 9, 12, and 24 h, respectively, and 4TZ in the bottom two rows (**i**–**p**) at 0, 0.5, 1, 3, 6, 9, 12, and 24 h, respectively, during immersion in 0.1 M NaCl.

Figure 7 shows optical microscopy images of TZ (top two rows, a–h) and 4TZ (bottom two rows, i–p) during immersion in 0.1 M NaCl over a 24-h period. In TZ, minimal surface changes were observed during the first 3 h (a)–(d)), except for the appearance of hydrogen bubbles. Small areas of corrosion were more prominent after 6 h (e) and increased at 9 h (f) and 12 h (g). After 24 h, a widespread distribution of corrosion spots was observed across the surface. In contrast, for 4TZ after 3 h of immersion (l), the entire surface darkened along with some hydrogen evolution, and by 6 h (m), large corrosion areas were visible. These areas expanded further at 9 h (n) and 12 h (o), culminating in extensive corrosion coverage by 24 h (p), with large patches and interconnected corrosion regions across the surface, accompanied by extensive hydrogen evolution.

The PDP curves revealed that both the 4TZ and TZ alloys were more corrosion resistant than pure Mg. Both PDP and optical microscopy during immersion tests indicated that TZ was more corrosion resistant than the 4TZ alloy. The presence of Ti_2_Zn in the TZ alloys could be attributed to the increased corrosion resistance. Şuctic et al. demonstrated that Ti rich intermetallics in Zn alloys act as corrosion barriers, stabilizing the surrounding matrix [13]. Similarly, Ti_2_Zn may have helped stabilize the Mg matrix by acting as a barrier, probably forming inert islands of TiO_x_, slowing the propagation of corrosion fronts, while also promoting localized anodic dissolution of Mg due to galvanic interactions. This localized effect, along with the distribution of Ti rich phases around micropores, may have created cathodic sites, leading to variations in corrosion susceptibility across the alloy.

### 3.4. Surface Characterization Using X-Ray Photoelectron Spectroscopy

**Figure 8:**
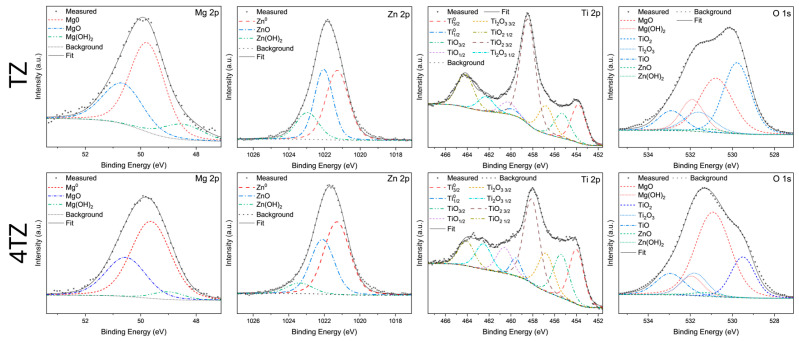
This figure shows the X-ray photoelectron spectroscopy results of TZ (top row) and 4TZ (bottom row) after 5 min of immersion in 0.1 M NaCl.

The results of X-ray photoelectron spectroscopy (XPS) after 5 min of immersion in 0.1 M NaCl are presented in Figure 8, with the TZ spectra in the top row and the 4TZ spectra in the bottom row. Both sets of high-resolution spectra include peaks corresponding to Mg 2p, Zn 2p, Ti 2p, and O 1s. For the TZ sample, the Mg 2p peak showed distinct components at lower binding energies, with contributions from Mg(OH)_2_ and MgO, while the majority of the spectrum was dominated by the bare metal peak (Mg^0^) at 58.04 at.%, with MgO accounting for 30.40 at.%. The Zn 2p spectrum revealed that the bare metal was more prevalent than the oxide, with 45.35 at.% Zn^0^ and 37.21 at.% ZnO. The Ti 2p spectrum indicated the presence of both TiO_2_ and lower-valence titanium oxides, with the oxide peaks comprising the majority of the scan at 83.97 at.%, while bare Ti contributed 16.03 at.%. The O 1s spectrum displayed components corresponding to hydroxides and oxides, with Ti oxides comprising the majority of the detected species at 52.90 at.%, followed by MgO at 31.87 at.%.

For 4TZ, the Mg 2p peak revealed contributions from Mg(OH)_2_ and MgO, with Mg^0^ at 62.70 at.% and MgO at 32.30 at.%. The Zn 2p spectrum shows that bare Zn was the primary species at 54.28 at.%, followed by ZnO at 37.82 at.%. The Ti 2p spectrum of 4TZ showed the presence of TiO_2_ and other oxides, where the oxide species accounted for 79.51 at.% of the spectrum, with bare Ti at 20.49 at.%. The O 1s spectrum was dominated by MgO at 51.69 at.%, with Ti oxides comprising 38.19 at.%.

In summary, both alloys developed surface films dominated by oxidized Ti species, while the presence of metallic elements was attributed to either a thinner film or unoxidized components trapped within the surface layer. The TZ alloy exhibited a higher concentration of TiO_2_, suggesting more effective surface passivation and improved corrosion resistance. In contrast, 4TZ exhibited a greater presence of bare Mg^0^ and MgO, indicating that Mg played a more dominant role in the surface reactions. Overall, TiO_2_ contributed substantially to surface stabilization in TZ, while Mg remained the primary active element driving the corrosion behavior in both alloys.

### 3.5. General Discussion

**Figure 9:**
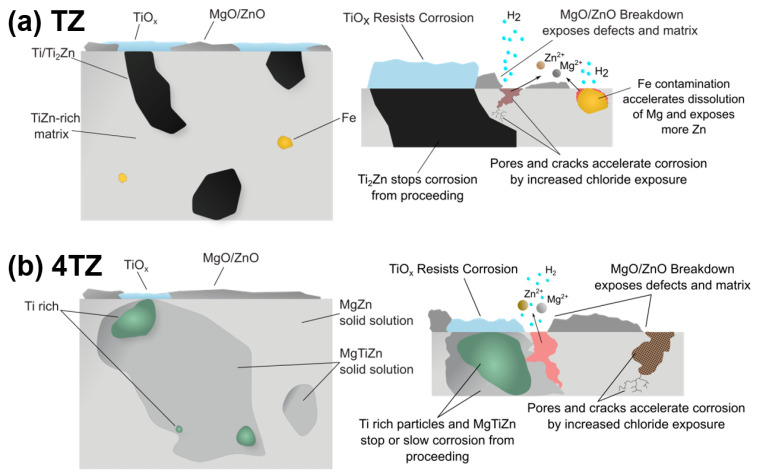
Schematic cross-sections of (**a**) TZ and (**b**) 4TZ alloys, illustrating the key microstructural features and proposed corrosion behavior. The TZ alloy contains Ti_2_Zn intermetallic phases, TiO_x_ oxides, MgO oxide, and dispersed Fe-rich inclusions within a TiZn matrix. In contrast, the 4TZ alloy exhibited larger Ti rich regions, distinct MgZn and MgTiZn solid solution domains, and similar oxide features.

The MgTiZn (TZ) and Mg_4_TiZn (4TZ) alloys developed in this study demonstrated a rare combination of high hardness, elastic modulus, and corrosion resistance, which are often mutually exclusive in Mg-based systems. As illustrated in the schematic cross-sections (Figure 9), the TZ alloy exhibited a microstructure containing abundant intermetallic phases (Ti_2_Zn and Mg_21_Zn_25_) within a Ti- and Zn-enriched matrix (confirmed by the STEM analysis) and fine oxide particles (TiO_x_ and MgO). In contrast, 4TZ presented a more homogeneous Mg-rich matrix containing solid solution domains composed of Mg, Ti, and Zn, and larger Ti rich regions. The presence of Ti and Zn in the matrix, along with uniformly distributed fine intermetallics, promoted the formation of a protective surface film (observed via XPS), leading to passivation and high corrosion resistance.

Although micropores were observed in both alloys (Figure 1), which are typically expected to increase corrosion susceptibility, the corrosion current densities of the alloys remained low, suggesting that the protective effect of passivating surface films and refined microstructures outweighed the detrimental influence of porosity. Nonetheless, minimizing porosity remains important, and future optimization of the SPS parameters may further improve the corrosion performance.

The observed high hardness and elastic modulus can be attributed to the synergy of phase selection and severe plastic deformation induced by ball milling. Even with a significantly higher Mg content, 4TZ maintained mechanical properties comparable to those of TZ due to defect-assisted strengthening and oxide dispersion. Meanwhile, the corrosion resistance, particularly in the TZ, was enhanced by the formation of stable Ti rich intermetallics and surface TiO_2_, which acted as passive barriers and limited corrosion front propagation.

The corrosion behavior of both alloys was primarily governed by the anodic dissolution of Mg, accompanied by the formation and partial breakdown of mixed oxide films. The anticipated electrochemical reactions in a neutral NaCl solution include:

$$\mathrm{Anodic}:\;Mg\to {Mg}^{2+}+2e^{-}$$

$$Zn\to {Zn}^{2+}+2e^{-}$$

$$\mathrm{Cathodic}:\;2H_{2}O+2e^{-}\to H_{2}+2OH^{-}$$

The dominant anodic reaction was Mg dissolution, which was consistent with the observed hydrogen evolution. Ti remains largely passive due to the formation of a stable TiO_2_ layer, while Zn may act both anodically and cathodically depending on the local environment and oxide stability.

Initially, a mixed surface film of MgO, ZnO, and TiO_2_ was formed during immersion. This film passivates portions of the surface, but breakdown occurs at weak points, especially near pores or compositional heterogeneities. Localized Mg dissolution initiates where the film fails, and Fe-rich inclusions may accelerate this process by acting as cathodic sites. Ti_2_Zn intermetallics act as partial barriers to corrosion front propagation, although they may also participate in galvanic coupling. In contrast, the more uniform phase distribution in 4TZ leads to slower localized degradation.

This model represents one possible interpretation of corrosion behavior based on the current data. Future work using advanced surface-sensitive techniques, such as angle-resolved XPS, SIMS, or high-resolution cross-sectional TEM, could reveal the detailed structure and layering of these oxide films and validate the assumptions underlying this proposed mechanism.

While qualitative phase identification confirmed the presence of Ti_2_Zn in TZ and its absence in 4TZ, this study did not conduct a quantitative phase analysis (e.g., area fraction or volume percent of Ti_2_Zn). Such an analysis would help establish a more direct correlation between the phase distribution and corrosion resistance, and will be the focus of future work. Additionally, although 4TZ lacked detectable Ti_2_Zn, it still exhibited good corrosion resistance, which may be attributed to a more uniform elemental distribution of Ti and Zn in the Mg-rich matrix and the presence of MgO, both of which could contribute to reduced microgalvanic activity and enhanced surface stability. The higher TiO_2_ concentration in TZ, observed via XPS, likely contributed to the greater passivation efficiency and lower corrosion current densities.

Although MgO was more prominent in 4TZ, its impact on corrosion was not straightforward. The conventional understanding suggests that oxides and other electrochemical heterogeneities generally promote localized corrosion. However, recent studies have indicated that the size, distribution, and morphology of such heterogeneities are critical [48,49]. In this case, MgO may be finely dispersed or exist in a form that does not significantly promote galvanic coupling. Additionally, oxygen can be partially incorporated as an interstitial solid solution, which has been suggested to enhance the passivation behavior in some systems [50]. Further work is required to elucidate the influence of oxygen incorporation on the corrosion resistance of these alloys.

These results underscore the potential of combining mechanical alloying and spark plasma sintering to tailor microstructures that balance the strength, stiffness, and corrosion behavior of Mg alloys. By leveraging solid-solution strengthening, intermetallic phase design, and controlled oxide formation, this approach opens a pathway toward multi-functional Mg-based materials suitable for structural or biomedical applications.

## 4. Conclusions

This study investigated the effects of composition and processing on the microstructure, hardness, elastic modulus, and corrosion resistance of spark plasma sintered Mg-Ti-Zn alloys. Key findings include:

- MgTiZn and Mg_4_TiZn alloys were produced via ball milling and spark plasma sintering. XRD, SEM, and STEM analyses revealed the presence of multiple phases.
- Both TZ and 4TZ exhibited high hardness and stiffness, which were much higher than those of any commercial Mg alloy.
- Both TZ and 4TZ exhibited high corrosion resistance (indicated by the high corrosion potential and low corrosion current density) and passivation, which were attributed to the formation of a Ti rich surface film.

These findings demonstrate the potential of combining mechanical alloying and SPS to engineer Mg-containing multi-principal element alloys with a rare combination of high hardness and corrosion resistance. This strategy offers a new pathway for designing lightweight structural or biomedical materials that overcome the traditional strength–corrosion trade-off in Mg and Zn alloys. In particular, this work lays the foundation for the future development of Zn and Mg-based alloys with improved mechanical performance for use in environments requiring both biocompatibility and corrosion resistance.

## Figures and Tables

**Table 1:** Composition of alloys.

Alloy	Mg wt.%	Ti wt.%	Zn wt.%
MgTiZn “TZ”	17.7	34.8	47.5
Mg_4_TiZn “4TZ”	46.2	22.7	31.1

**Table 2:** Summary of mechanical properties of TZ, 4TZ, and selected Zn, Ti, and Mg-based alloys from literature.

Alloy	Hardness (GPa)	Young’s Modulus (GPa)	Refs.
TZ	5.47 ± 1.02	98.18 ± 11.15	This work
4TZ	5.05 ± 0.89	100.75 ± 9.63	This work
Zn-(0.05–0.3)Ti	0.41–0.58	-	[11]
Zn-(0.1–1)Ti	0.33–0.69	-	[13]
Ti-(5–30)Zn	1.5–3.8	4.3–26.8	[12]
ZK60 (Mg-5.5Zn)	0.69	-	[44]
AZ31	0.59	44.8	[40,41]
WE43C	0.93	44.0	[42,43]

**Table 3:** Corrosion potentials and current densities of TZ, 4TZ, and comparable Zn, Ti, and Mg-based alloys from the literature.

Alloy	E_corr_ (mV_SCE_)	i_corr_ (µA/cm^2^)	Solution	Refs.
TZ	−972 ± 78	3.65 ± 0.65	0.1 M NaCl	This work
4TZ	−1078 ± 2	4.58 ± 1.64	0.1 M NaCl	This work
Zn-0.05Ti	−1036 ± 78	19.7 ± 0.4	HBSS	[11]
Zn-0.1Ti	−1038 ± 94	21.6 ± 0.6	HBSS	[11]
Zn-0.2Ti	−1045 ± 72	22.8 ± 0.7	HBSS	[11]
Zn-0.3Ti	−1049 ± 82	27.4 ± 0.3	HBSS	[11]
Zn-0.10Ti	−1005.6	33.4	0.9% NaCl saline	[13]
Zn-0.25Ti	−1035.3	16.2	0.9% NaCl saline	[13]
Zn-1.00Ti	−1048.4	15.6	0.9% NaCl saline	[13]
Ti-5Zn	−187	0.69	SBF	[12]
Ti-10Zn	−202	0.97	SBF	[12]
Ti-20Zn	−178	0.74	SBF	[12]
Ti-30Zn	−245	3.63	SBF	[12]
ZA-8	−1050	127.54	0.6 M NaCl	[45]
ZK60 (Mg-5.5Zn)	−1500	50.4	3 wt.% NaCl	[44]
AZ31 (Mg-3Al-1Zn)	−1483	154.03	0.1 M NaCl	[46]
WE43C	−1720 ± 20	65.7 ± 2	0.6 M NaCl	[47]

## Data Availability

The original contributions presented in this study are included in the article. Further inquiries can be directed to the corresponding authors.

## Abbreviations

The following abbreviations are used in this manuscript:

BCC	Body-centered cubic
BCT	Body-centered tetragonal
EDXS	Energy-dispersive X-ray spectroscopy
FCC	Face-centered cubic
FIB	Focused ion beam
HAADF	High-angle annular dark-field
HEBM	High-energy ball milling
HCP	Hexagonal close-packed
OCP	Open circuit potential
PDP	Potentiodynamic polarization
RPM	Revolutions per minute
SAED	Selected area electron diffraction
SCE	Saturated calomel electrode
SEM	Scanning electron microscopy
SPS	Spark plasma sintering
STEM	Scanning transmission electron microscopy
TEM	Transmission electron microscopy
TZ	Equal molar MgTiZn alloy
4TZ	Mg_4_TiZn alloy
XPS	X-ray photoelectron spectroscopy
XRD	X-ray diffraction
MPEA	Multi-principle element alloy
